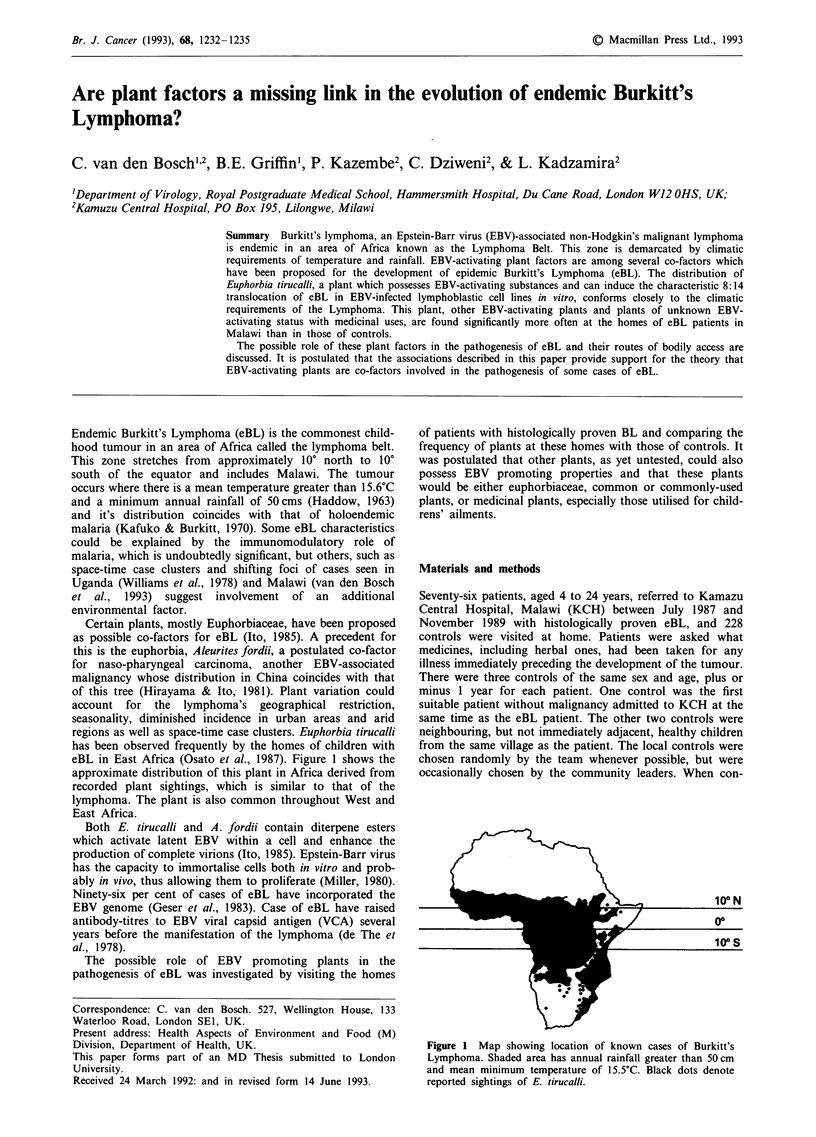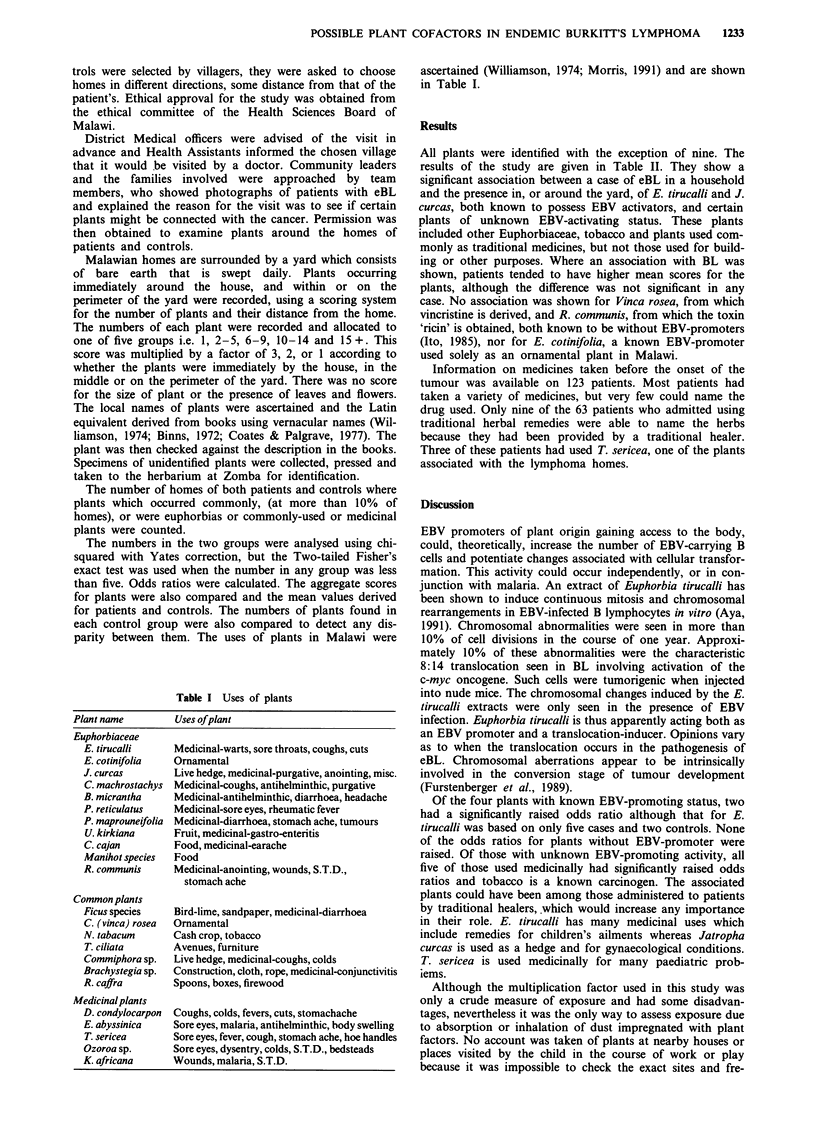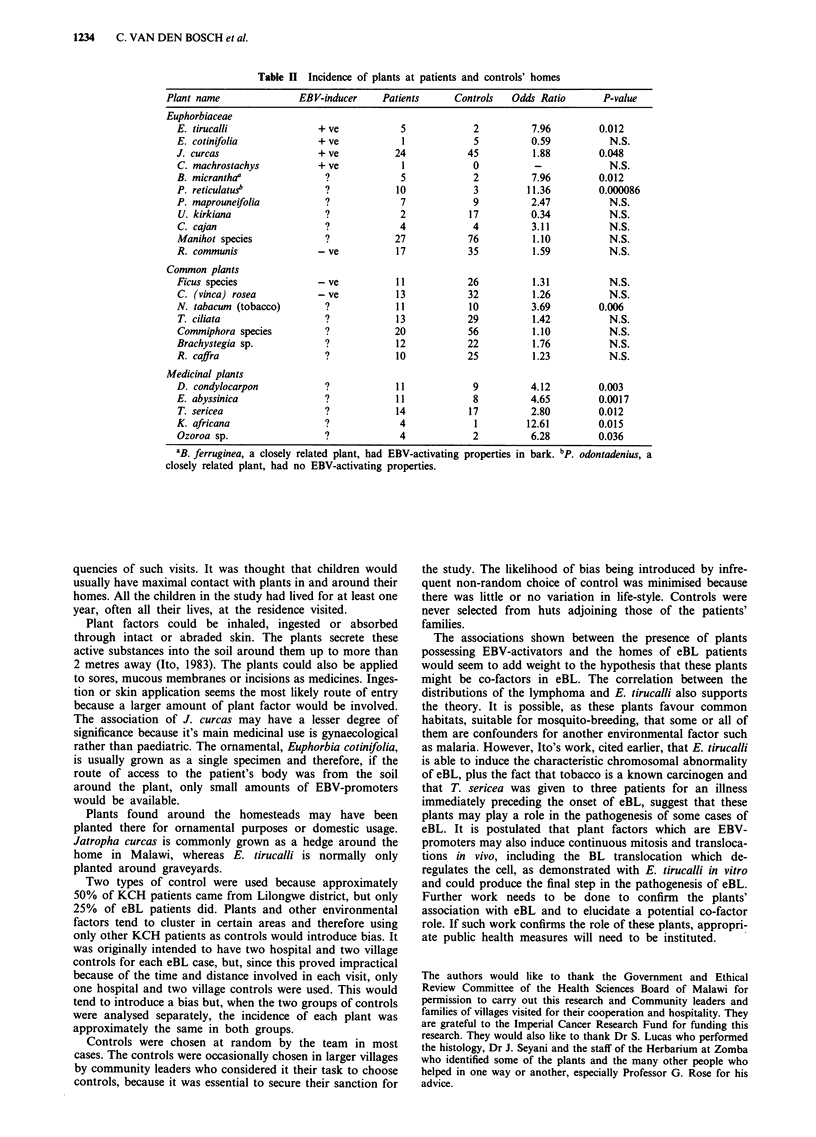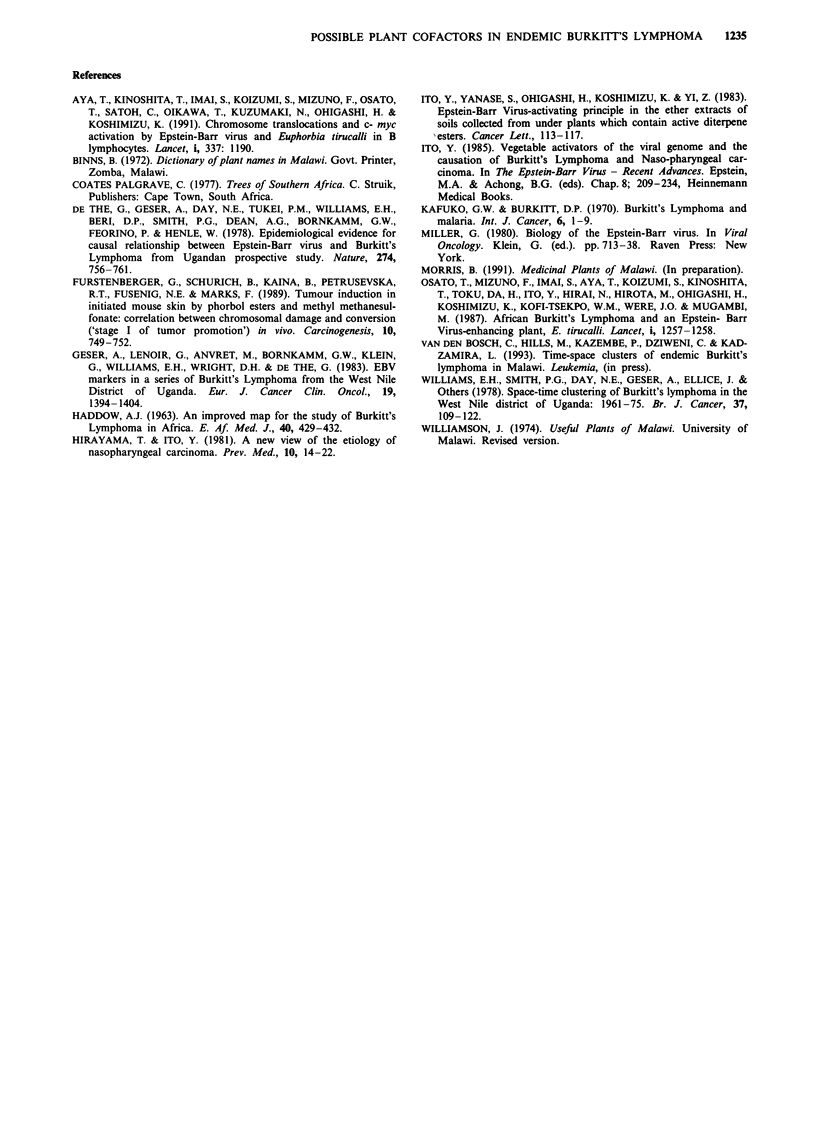# Are plant factors a missing link in the evolution of endemic Burkitt's lymphoma?

**DOI:** 10.1038/bjc.1993.510

**Published:** 1993-12

**Authors:** C. van den Bosch, B. E. Griffin, P. Kazembe, C. Dziweni, L. Kadzamira

**Affiliations:** Department of Virology, Royal Postgraduate Medical School, Hammersmith Hospital, London, UK.

## Abstract

Burkitt's lymphoma, an Epstein-Barr virus (EBV)-associated non-Hodgkin's malignant lymphoma is endemic in an area of Africa known as the Lymphoma Belt. This zone is demarcated by climatic requirements of temperature and rainfall. EBV-activating plant factors are among several co-factors which have been proposed for the development of epidemic Burkitt's Lymphoma (eBL). The distribution of Euphorbia tirucalli, a plant which possesses EBV-activating substances and can induce the characteristic 8:14 translocation of eBL in EBV-infected lymphoblastic cell lines in vitro, conforms closely to the climatic requirements of the Lymphoma. This plant, other EBV-activating plants and plants of unknown EBV-activating status with medicinal uses, are found significantly more often at the homes of eBL patients in Malawi than in those of controls. The possible role of these plant factors in the pathogenesis of eBL and their routes of bodily access are discussed. It is postulated that the associations described in this paper provide support for the theory that EBV-activating plants are co-factors involved in the pathogenesis of some cases of eBL.


Br. J. Cancer (1993), 68, 1232–1235 © Macmillan Press Ltd., 1993

### Are plant factors a missing link in the evolution of endemic Burkitt's Lymphoma?

C. van den Bosch[1,2], B.E. Griffin[1], P. Kazembe[2], C. Dziweni[2], & L. Kadzamira[2]

[1]*Department of Virology, Royal Postgraduate Medical School, Hammersmith Hospital, Du Cane Road, London W12 0HS, UK;* [2]*Kamuzu Central Hospital, PO Box 195, Lilongwe, Milawi*

**Summary** Burkitt's lymphoma, an Epstein-Barr virus (EBV)-associated non-Hodgkin's malignant lymphoma is endemic in an area of Africa known as the Lymphoma Belt. This zone is demarcated by climatic requirements of temperature and rainfall. EBV-activating plant factors are among several co-factors which have been proposed for the development of epidemic Burkitt's Lymphoma (eBL). The distribution of *Euphorbia tirucalli*, a plant which possesses EBV-activating substances and can induce the characteristic 8:14 translocation of eBL in EBV-infected lymphoblastic cell lines *in vitro*, conforms closely to the climatic requirements of the Lymphoma. This plant, other EBV-activating plants and plants of unknown EBV-activating status with medicinal uses, are found significantly more often at the homes of eBL patients in Malawi than in those of controls.

The possible role of these plant factors in the pathogenesis of eBL and their routes of bodily access are discussed. It is postulated that the associations described in this paper provide support for the theory that EBV-activating plants are co-factors involved in the pathogenesis of some cases of eBL.

Endemic Burkitt's Lymphoma (eBL) is the commonest childhood tumour in an area of Africa called the lymphoma belt. This zone stretches from approximately 10° north to 10° south of the equator and includes Malawi. The tumour occurs where there is a mean temperature greater than 15.6°C and a minimum annual rainfall of 50 cms (Haddow, 1963) and it's distribution coincides with that of holoendemic malaria (Kafuko & Burkitt, 1970). Some eBL characteristics could be explained by the immunomodulatory role of malaria, which is undoubtedly significant, but others, such as space-time case clusters and shifting foci of cases seen in Uganda (Williams *et al.*, 1978) and Malawi (van den Bosch *et al.*, 1993) suggest involvement of an additional environmental factor.

Certain plants, mostly Euphorbiaceae, have been proposed as possible co-factors for eBL (Ito, 1985). A precedent for this is the euphorbia, *Aleurites fordii*, a postulated co-factor for naso-pharyngeal carcinoma, another EBV-associated malignancy whose distribution in China coincides with that of this tree (Hirayama & Ito, 1981). Plant variation could account for the lymphoma's geographical restriction, seasonality, diminished incidence in urban areas and arid regions as well as space-time case clusters. *Euphorbia tirucalli* has been observed frequently by the homes of children with eBL in East Africa (Osato *et al.*, 1987). Figure 1 shows the approximate distribution of this plant in Africa derived from recorded plant sightings, which is similar to that of the lymphoma. The plant is also common throughout West and East Africa.

Both *E. tirucalli* and *A. fordii* contain diterpene esters which activate latent EBV within a cell and enhance the production of complete virions (Ito, 1985). Epstein-Barr virus has the capacity to immortalise cells both *in vitro* and probably *in vivo*, thus allowing them to proliferate (Miller, 1980). Ninety-six per cent of cases of eBL have incorporated the EBV genome (Geser *et al.*, 1983). Case of eBL have raised antibody-titres to EBV viral capsid antigen (VCA) several years before the manifestation of the lymphoma (de The *et al.*, 1978).

The possible role of EBV promoting plants in the pathogenesis of eBL was investigated by visiting the homes of patients with histologically proven BL and comparing the frequency of plants at these homes with those of controls. It was postulated that other plants, as yet untested, could also possess EBV promoting properties and that these plants would be either euphorbiaceae, common or commonly-used plants, or medicinal plants, especially those utilised for childrens' ailments.

### Materials and methods

Seventy-six patients, aged 4 to 24 years, referred to Kamazu Central Hospital, Malawi (KCH) between July 1987 and November 1989 with histologically proven eBL, and 228 controls were visited at home. Patients were asked what medicines, including herbal ones, had been taken for any illness immediately preceding the development of the tumour. There were three controls of the same sex and age, plus or minus 1 year for each patient. One control was the first suitable patient without malignancy admitted to KCH at the same time as the eBL patient. The other two controls were neighbouring, but not immediately adjacent, healthy children from the same village as the patient. The local controls were chosen randomly by the team whenever possible, but were occasionally chosen by the community leaders. When con-

**Figure 1** Map showing location of known cases of Burkitt's Lymphoma. Shaded area has annual rainfall greater than 50 cm and mean minimum temperature of 15.5°C. Black dots denote reported sightings of *E. tirucalli.*

Correspondence: C. van den Bosch. 527, Wellington House, 133 Waterloo Road, London SE1, UK.
Present address: Health Aspects of Environment and Food (M) Division, Department of Health, UK.
This paper forms part of an MD Thesis submitted to London University.
Received 24 March 1992: and in revised form 14 June 1993.

trols were selected by villagers, they were asked to choose homes in different directions, some distance from that of the patient's. Ethical approval for the study was obtained from the ethical committee of the Health Sciences Board of Malawi.

District Medical officers were advised of the visit in advance and Health Assistants informed the chosen village that it would be visited by a doctor. Community leaders and the families involved were approached by team members, who showed photographs of patients with eBL and explained the reason for the visit was to see if certain plants might be connected with the cancer. Permission was then obtained to examine plants around the homes of patients and controls.

Malawian homes are surrounded by a yard which consists of bare earth that is swept daily. Plants occurring immediately around the house, and within or on the perimeter of the yard were recorded, using a scoring system for the number of plants and their distance from the home. The numbers of each plant were recorded and allocated to one of five groups i.e. 1, 2–5, 6–9, 10–14 and 15+. This score was multiplied by a factor of 3, 2, or 1 according to whether the plants were immediately by the house, in the middle or on the perimeter of the yard. There was no score for the size of plant or the presence of leaves and flowers. The local names of plants were ascertained and the Latin equivalent derived from books using vernacular names (Williamson, 1974; Binns, 1972; Coates & Palgrave, 1977). The plant was then checked against the description in the books. Specimens of unidentified plants were collected, pressed and taken to the herbarium at Zomba for identification.

The number of homes of both patients and controls where plants which occurred commonly, (at more than 10% of homes), or were euphorbias or commonly-used or medicinal plants were counted.

The numbers in the two groups were analysed using chi-squared with Yates correction, but the Two-tailed Fisher's exact test was used when the number in any group was less than five. Odds ratios were calculated. The aggregate scores for plants were also compared and the mean values derived for patients and controls. The numbers of plants found in each control group were also compared to detect any disparity between them. The uses of plants in Malawi were ascertained (Williamson, 1974; Morris, 1991) and are shown in Table I.

**Table I** Uses of plants

| *Plant name* | *Uses of plant* |
|---|---|
| *Euphorbiaceae* | |
| *E. tirucalli* | Medicinal-warts, sore throats, coughs, cuts |
| *E. cotinifolia* | Ornamental |
| *J. curcas* | Live hedge, medicinal-purgative, anointing, misc. |
| *C. machrostachys* | Medicinal-coughs, antihelminthic, purgative |
| *B. micrantha* | Medicinal-antihelminthic, diarrhoea, headache |
| *P. reticulatus* | Medicinal-sore eyes, rheumatic fever |
| *P. maprouneifolia* | Medicinal-diarrhoea, stomach ache, tumours |
| *U. kirkiana* | Fruit, medicinal-gastro-enteritis |
| *C. cajan* | Food, medicinal-earache |
| *Manihot species* | Food |
| *R. communis* | Medicinal-anointing, wounds, S.T.D., stomach ache |
| *Common plants* | |
| *Ficus* species | Bird-lime, sandpaper, medicinal-diarrhoea |
| *C. (vinca) rosea* | Ornamental |
| *N. tabacum* | Cash crop, tobacco |
| *T. ciliata* | Avenues, furniture |
| *Commiphora* sp. | Live hedge, medicinal-coughs, colds |
| *Brachystegia* sp. | Construction, cloth, rope, medicinal-conjunctivitis |
| *R. caffra* | Spoons, boxes, firewood |
| *Medicinal plants* | |
| *D. condylocarpon* | Coughs, colds, fevers, cuts, stomachache |
| *E. abyssinica* | Sore eyes, malaria, antihelminthic, body swelling |
| *T. sericea* | Sore eyes, fever, cough, stomach ache, hoe handles |
| *Ozoroa* sp. | Sore eyes, dysentry, colds, S.T.D., bedsteads |
| *K. africana* | Wounds, malaria, S.T.D. |

### Results

All plants were identified with the exception of nine. The results of the study are given in Table II. They show a significant association between a case of eBL in a household and the presence in, or around the yard, of *E. tirucalli* and *J. curcas*, both known to possess EBV activators, and certain plants of unknown EBV-activating status. These plants included other Euphorbiaceae, tobacco and plants used commonly as traditional medicines, but not those used for building or other purposes. Where an association with BL was shown, patients tended to have higher mean scores for the plants, although the difference was not significant in any case. No association was shown for *Vinca rosea*, from which vincristine is derived, and *R. communis*, from which the toxin 'ricin' is obtained, both known to be without EBV-promoters (Ito, 1985), nor for *E. cotinifolia*, a known EBV-promoter used solely as an ornamental plant in Malawi.

Information on medicines taken before the onset of the tumour was available on 123 patients. Most patients had taken a variety of medicines, but very few could name the drug used. Only nine of the 63 patients who admitted using traditional herbal remedies were able to name the herbs because they had been provided by a traditional healer. Three of these patients had used *T. sericea*, one of the plants associated with the lymphoma homes.

### Discussion

EBV promoters of plant origin gaining access to the body, could, theoretically, increase the number of EBV-carrying B cells and potentiate changes associated with cellular transformation. This activity could occur independently, or in conjunction with malaria. An extract of *Euphorbia tirucalli* has been shown to induce continuous mitosis and chromosomal rearrangements in EBV-infected B lymphocytes *in vitro* (Aya, 1991). Chromosomal abnormalities were seen in more than 10% of cell divisions in the course of one year. Approximately 10% of these abnormalities were the characteristic 8:14 translocation seen in BL involving activation of the c-*myc* oncogene. Such cells were tumorigenic when injected into nude mice. The chromosomal changes induced by the *E. tirucalli* extracts were only seen in the presence of EBV infection. *Euphorbia tirucalli* is thus apparently acting both as an EBV promoter and a translocation-inducer. Opinions vary as to when the translocation occurs in the pathogenesis of eBL. Chromosomal aberrations appear to be intrinsically involved in the conversion stage of tumour development (Furstenberger *et al.*, 1989).

Of the four plants with known EBV-promoting status, two had a significantly raised odds ratio although that for *E. tirucalli* was based on only five cases and two controls. None of the odds ratios for plants without EBV-promoter were raised. Of those with unknown EBV-promoting activity, all five of those used medicinally had significantly raised odds ratios and tobacco is a known carcinogen. The associated plants could have been among those administered to patients by traditional healers, which would increase any importance in their role. *E. tirucalli* has many medicinal uses which include remedies for children's ailments whereas *Jatropha curcas* is used as a hedge and for gynaecological conditions. *T. sericea* is used medicinally for many paediatric problems.

Although the multiplication factor used in this study was only a crude measure of exposure and had some disadvantages, nevertheless it was the only way to assess exposure due to absorption or inhalation of dust impregnated with plant factors. No account was taken of plants at nearby houses or places visited by the child in the course of work or play because it was impossible to check the exact sites and fre-

**Table II** Incidence of plants at patients and controls' homes

| *Plant name* | *EBV-inducer* | *Patients* | *Controls* | *Odds Ratio* | *P-value* |
|---|---|---|---|---|---|
| *Euphorbiaceae* | | | | | |
| *E. tirucalli* | + ve | 5 | 2 | 7.96 | 0.012 |
| *E. cotinifolia* | + ve | 1 | 5 | 0.59 | N.S. |
| *J. curcas* | + ve | 24 | 45 | 1.88 | 0.048 |
| *C. machrostachys* | + ve | 1 | 0 | – | N.S. |
| *B. micrantha*[a] | ? | 5 | 2 | 7.96 | 0.012 |
| *P. reticulatus*[b] | ? | 10 | 3 | 11.36 | 0.000086 |
| *P. maprouneifolia* | ? | 7 | 9 | 2.47 | N.S. |
| *U. kirkiana* | ? | 2 | 17 | 0.34 | N.S. |
| *C. cajan* | ? | 4 | 4 | 3.11 | N.S. |
| *Manihot* species | ? | 27 | 76 | 1.10 | N.S. |
| *R. communis* | – ve | 17 | 35 | 1.59 | N.S. |
| *Common plants* | | | | | |
| *Ficus* species | – ve | 11 | 26 | 1.31 | N.S. |
| *C. (vinca) rosea* | – ve | 13 | 32 | 1.26 | N.S. |
| *N. tabacum* (tobacco) | ? | 11 | 10 | 3.69 | 0.006 |
| *T. ciliata* | ? | 13 | 29 | 1.42 | N.S. |
| *Commiphora* species | ? | 20 | 56 | 1.10 | N.S. |
| *Brachystegia* sp. | ? | 12 | 22 | 1.76 | N.S. |
| *R. caffra* | ? | 10 | 25 | 1.23 | N.S. |
| *Medicinal plants* | | | | | |
| *D. condylocarpon* | ? | 11 | 9 | 4.12 | 0.003 |
| *E. abyssinica* | ? | 11 | 8 | 4.65 | 0.0017 |
| *T. sericea* | ? | 14 | 17 | 2.80 | 0.012 |
| *K. africana* | ? | 4 | 1 | 12.61 | 0.015 |
| *Ozoroa* sp. | ? | 4 | 2 | 6.28 | 0.036 |

[a]*B. ferruginea*, a closely related plant, had EBV-activating properties in bark. [b]*P. odontadenius*, a closely related plant, had no EBV-activating properties.

quencies of such visits. It was thought that children would usually have maximal contact with plants in and around their homes. All the children in the study had lived for at least one year, often all their lives, at the residence visited.

Plant factors could be inhaled, ingested or absorbed through intact or abraded skin. The plants secrete these active substances into the soil around them up to more than 2 metres away (Ito, 1983). The plants could also be applied to sores, mucous membranes or incisions as medicines. Ingestion or skin application seems the most likely route of entry because a larger amount of plant factor would be involved. The association of *J. curcas* may have a lesser degree of significance because it's main medicinal use is gynaecological rather than paediatric. The ornamental, *Euphorbia cotinifolia*, is usually grown as a single specimen and therefore, if the route of access to the patient's body was from the soil around the plant, only small amounts of EBV-promoters would be available.

Plants found around the homesteads may have been planted there for ornamental purposes or domestic usage. *Jatropha curcas* is commonly grown as a hedge around the home in Malawi, whereas *E. tirucalli* is normally only planted around graveyards.

Two types of control were used because approximately 50% of KCH patients came from Lilongwe district, but only 25% of eBL patients did. Plants and other environmental factors tend to cluster in certain areas and therefore using only other KCH patients as controls would introduce bias. It was originally intended to have two hospital and two village controls for each eBL case, but, since this proved impractical because of the time and distance involved in each visit, only one hospital and two village controls were used. This would tend to introduce a bias but, when the two groups of controls were analysed separately, the incidence of each plant was approximately the same in both groups.

Controls were chosen at random by the team in most cases. The controls were occasionally chosen in larger villages by community leaders who considered it their task to choose controls, because it was essential to secure their sanction for the study. The likelihood of bias being introduced by infrequent non-random choice of control was minimised because there was little or no variation in life-style. Controls were never selected from huts adjoining those of the patients' families.

The associations shown between the presence of plants possessing EBV-activators and the homes of eBL patients would seem to add weight to the hypothesis that these plants might be co-factors in eBL. The correlation between the distributions of the lymphoma and *E. tirucalli* also supports the theory. It is possible, as these plants favour common habitats, suitable for mosquito-breeding, that some or all of them are confounders for another environmental factor such as malaria. However, Ito's work, cited earlier, that *E. tirucalli* is able to induce the characteristic chromosomal abnormality of eBL, plus the fact that tobacco is a known carcinogen and that *T. sericea* was given to three patients for an illness immediately preceding the onset of eBL, suggest that these plants may play a role in the pathogenesis of some cases of eBL. It is postulated that plant factors which are EBV-promoters may also induce continuous mitosis and translocations *in vivo*, including the BL translocation which deregulates the cell, as demonstrated with *E. tirucalli in vitro* and could produce the final step in the pathogenesis of eBL. Further work needs to be done to confirm the plants' association with eBL and to elucidate a potential co-factor role. If such work confirms the role of these plants, appropriate public health measures will need to be instituted.

The authors would like to thank the Government and Ethical Review Committee of the Health Sciences Board of Malawi for permission to carry out this research and Community leaders and families of villages visited for their cooperation and hospitality. They are grateful to the Imperial Cancer Research Fund for funding this research. They would also like to thank Dr S. Lucas who performed the histology, Dr J. Seyani and the staff of the Herbarium at Zomba who identified some of the plants and the many other people who helped in one way or another, especially Professor G. Rose for his advice.

**References**

AYA, T., KINOSHITA, T., IMAI, S., KOIZUMI, S., MIZUNO, F., OSATO, T., SATOH, C., OIKAWA, T., KUZUMAKI, N., OHIGASHI, H. & KOSHIMIZU, K. (1991). Chromosome translocations and c- *myc* activation by Epstein-Barr virus and *Euphorbia tirucalli* in B lymphocytes. *Lancet*, **i**, 337: 1190.

BINNS, B. (1972). *Dictionary of plant names in Malawi*. Govt. Printer, Zomba, Malawi.

COATES PALGRAVE, C. (1977). *Trees of Southern Africa*. C. Struik, Publishers: Cape Town, South Africa.

DE THE, G., GESER, A., DAY, N.E., TUKEI, P.M., WILLIAMS, E.H., BERI, D.P., SMITH, P.G., DEAN, A.G., BORNKAMM, G.W., FEORINO, P. & HENLE, W. (1978). Epidemiological evidence for causal relationship between Epstein-Barr virus and Burkitt's Lymphoma from Ugandan prospective study. *Nature*, **274**, 756-761.

FURSTENBERGER, G., SCHURICH, B., KAINA, B., PETRUSEVSKA, R.T., FUSENIG, N.E. & MARKS, F. (1989). Tumour induction in initiated mouse skin by phorbol esters and methyl methanesulfonate: correlation between chromosomal damage and conversion ('stage I of tumor promotion') *in vivo*. *Carcinogenesis*, **10**, 749-752.

GESER, A., LENOIR, G., ANVRET, M., BORNKAMM, G.W., KLEIN, G., WILLIAMS, E.H., WRIGHT, D.H. & DE THE, G. (1983). EBV markers in a series of Burkitt's Lymphoma from the West Nile District of Uganda. *Eur. J. Cancer Clin. Oncol.*, **19**, 1394-1404.

HADDOW, A.J. (1963). An improved map for the study of Burkitt's Lymphoma in Africa. *E. Af. Med. J.*, **40**, 429-432.

HIRAYAMA, T. & ITO, Y. (1981). A new view of the etiology of nasopharyngeal carcinoma. *Prev. Med.*, **10**, 14-22.

ITO, Y., YANASE, S., OHIGASHI, H., KOSHIMIZU, K. & YI, Z. (1983). Epstein-Barr Virus-activating principle in the ether extracts of soils collected from under plants which contain active diterpene esters. *Cancer Lett.*, 113-117.

ITO, Y. (1985). Vegetable activators of the viral genome and the causation of Burkitt's Lymphoma and Naso-pharyngeal carcinoma. In *The Epstein-Barr Virus - Recent Advances*. Epstein, M.A. & Achong, B.G. (eds). Chap. 8; 209-234, Heinnemann Medical Books.

KAFUKO, G.W. & BURKITT, D.P. (1970). Burkitt's Lymphoma and malaria. *Int. J. Cancer*, **6**, 1-9.

MILLER, G. (1980). Biology of the Epstein-Barr virus. In *Viral Oncology*. Klein, G. (ed.). pp. 713-38. Raven Press: New York.

MORRIS, B. (1991). *Medicinal Plants of Malawi*. (In preparation).

OSATO, T., MIZUNO, F., IMAI, S., AYA, T., KOIZUMI, S., KINOSHITA, T., TOKU, DA, H., ITO, Y., HIRAI, N., HIROTA, M., OHIGASHI, H., KOSHIMIZU, K., KOFI-TSEKPO, W.M., WERE, J.O. & MUGAMBI, M. (1987). African Burkitt's Lymphoma and an Epstein- Barr Virus-enhancing plant, *E. tirucalli*. *Lancet*, **i**, 1257-1258.

VAN DEN BOSCH, C., HILLS, M., KAZEMBE, P., DZIWENI, C. & KADZAMIRA, L. (1993). Time-space clusters of endemic Burkitt's lymphoma in Malawi. *Leukemia*, (in press).

WILLIAMS, E.H., SMITH, P.G., DAY, N.E., GESER, A., ELLICE, J. & Others (1978). Space-time clustering of Burkitt's lymphoma in the West Nile district of Uganda: 1961-75. *Br. J. Cancer*, **37**, 109-122.

WILLIAMSON, J. (1974). *Useful Plants of Malawi*. University of Malawi. Revised version.